# The Role of Musculoskeletal Ultrasound in Detecting Superior Cluneal Nerve Entrapment: Biomechanical Insights in Chronic Low Back Pain—A Pilot Study

**DOI:** 10.3390/diagnostics16030469

**Published:** 2026-02-03

**Authors:** Giovanni Iudicelli, Francesco Agostini, Alberto Altarocca, Francesco Ioppolo, Marco Narciso, Marco Conti, Andrea Fisicaro, Alessio Savina, Vincenzo Di Nunno, Massimiliano Mangone, Stefano Galletti, Marco Paoloni

**Affiliations:** 1 Department of Anatomy, Histology, Forensic Medicine and Orthopedics, Sapienza University, 00185 Rome, Italy; francesco.agostini@uniroma1.it (F.A.); marco.narciso@uniroma1.it (M.N.); ma.conti@uniroma1.it (M.C.); andrea.fisicaro@uniroma1.it (A.F.); alessio.savina@uniroma1.it (A.S.); massimiliano.mangone@uniroma1.it (M.M.); marco.paoloni@uniroma1.it (M.P.); 2 Rehabilitation Unit, University Hospital Umberto I, 00185 Rome, Italy; a.altarocca@policlinicoumberto1.it (A.A.); f.ioppolo@policlinicoumberto1.it (F.I.); 3 ULSS9 “Scaligera”, 37122 Verona, Italy; vincenzo.dinunno@aulss9.veneto.it; 4 Musculoskeletal Ultrasound School, Italian Society for Ultrasound in Medicine and Biology, 40124 Bologna, Italy; stefanogalletti2011@gmail.com

**Keywords:** superior cluneal nerve, chronic low back pain, musculoskeletal ultrasound, thoracolumbar fascia, Copeman nodules, iliac crest enthesophytes

## Abstract

**Background**: Superior cluneal nerve (SCN) entrapment is frequently underrecognized as a contributor to chronic Low Back Pain (cLBP) and gluteal pain. Musculoskeletal ultrasound may reveal surrogate markers indicative of a biomechanical entrapment environment. The primary objective was the prevalence of the ultrasound marker triad (Copeman Nod-ules-CN, thoracolumbar fascia-TLF thickening > 3 mm, and iliac enthesophytes. Secondary objectives included mean TLF thickness and its correlation with numeric pain rating scale (NPRS) and Douleur Neuropathique en 4 questions scores (DN4). **Methods**: In this single-center, cross-sectional observational pilot study, we enrolled 12 patients with cLBP (>12 weeks) localized to the SCN distribution and a healthy control group (12). Ultrasound measurements included TLF thickness in longitudinal and transverse planes, TLF convexity loss, iliac crest enthesophytes, and CN. Statistical analyses comprised Mann-Whitney U test, Fisher exact test, Spearman rank correlation, and multivariate logistic regression. Significance was set at *p* < 0.05. **Results**: The ultrasound marker triad (CN, iliac enthesophytes, and TLF thickening > 3 mm) demonstrated high diagnostic specificity: individually, CN were present in 91.7% of patients vs. 8.3% of controls (*p* < 0.001), iliac enthesophytes in 58.3% vs. 0% (*p* = 0.005), TLF thickening > 3 mm in 41.7% of patients vs. 0% of controls (*p* < 0.001)and TLF convexity loss in 100% vs. 75% (*p* = 0.03). Mean TLF thickness was significantly greater in patients—3.53 ± 0.46 mm longitudinal and 3.42 ± 0.39 mm transverse—compared with controls (2.61 ± 0.28 mm and 2.50 ± 0.32 mm; both *p* < 0.001). TLF thickness correlated strongly with NPRS (Spearman rho = 0.825; *p* = 0.001) but not with DN4. Logistic regression demonstrated that the marker triad accounted for 67% of NPRS variance (R^2^ = 0.67). **Conclusions**: Ultrasound-detected fascial alterations and enthesopathic changes act as reliable surrogate markers for SCN entrapment and correlate strongly with nociceptive pain severity. The absence of correlation with neuropathic pain scores suggests a predominant fascial-muscular biomechanical mechanism rather than direct nerve damage. Incorporating this non-invasive protocol into clinical practice may enhance diagnostic precision and inform targeted rehabilitative strategies. Future multicenter, prospective studies with larger cohorts are warranted to validate these findings and establish standardized ultrasound criteria.

## 1. Introduction

Chronic Low Back Pain (cLBP) represents a major global health burden, affecting millions of individuals worldwide and imposing substantial socioeconomic costs. Although many cases are attributed to degenerative disc disease, facet joint arthropathy, or sacroiliac dysfunction, a significant proportion remains undiagnosed or misdiagnosed [1]. Superior cluneal nerve (SCN) entrapment, occurring at the osteofibrous tunnel along the posterior iliac crest where the nerve pierces the thoracolumbar fascia (TLF), has emerged as an underrecognized yet clinically significant cause of cLBP and gluteal pain [1,2]. The SCN originates variably from the dorsal rami of T11 to L5, most commonly from L1–L3, and courses through the paraspinal musculature before piercing the TLF near the posterior iliac crest [3]. At this anatomical location, the nerve traverses osteo-fibrous tunnels formed by the fascia and iliac bone, predisposing it to mechanical compression. Cadaveric studies have documented the presence of such tunnels in over 50% of specimens, with the medial branch most frequently involved [3]. The estimated prevalence of SCN entrapment in cLBP populations ranges from 12% to 14% [1,2,3]. Clinically, SCN entrapment presents burning pain in the superior gluteal region, often radiating down the posterior thigh, mimicking radicular sciatica (“pseudo-sciatica”) in the absence of neurological deficits [4]. Tenderness along the posterior iliac crest, reproducible by palpation or sono-palpation, is highly suggestive. Diagnostic confirmation traditionally relies on clinical examination and response to anesthetic nerve blocks [4]. Despite growing clinical interest, diagnostic ultrasound protocols for SCN entrapment remain poorly standardized [5,6,7]. Most studies have focused on ultrasound-guided nerve blocks rather than diagnostic imaging [5,6,7]. Direct visualization of the SCN is challenging due to its small diameter (mean 1.1 mm), variable course, and patient-related factors such as obesity and tissue echogenicity [5]. Consequently, surrogate ultrasound markers may provide valuable diagnostic support [5,6,7]. Musculoskeletal ultrasound offers a non-invasive, dynamic, real-time, and cost-effective imaging modality for evaluating soft tissue and osseous structures in the lumbosacral region [5,6,7,8,9]. In the literature, there are recurrent patterns of specific ultrasound findings in patients presenting cLBP localized to the SCN distribution [9]. These included thoracolumbar fascia (TLF) thickening, iliac crest enthesophytes at the fascial insertion site, and subcutaneous Copeman nodules (CN) in the lumbar and iliac region. TLF is a richly innervated connective tissue structure with significant nociceptive potential due to A-delta and C-fiber innervation. Pathological TLF thickening, defined as exceeding 2 mm at the iliac insertion site on ultrasound measurement, is associated with reduced elasticity, diminished shear mobility with underlying musculature, and cLBP [8,9]. Recent studies further elaborate on this innervation, showing nociceptive fibers across fascial layers, with pathological increases in density and sensitization during inflammation or fibrosis. Ultrasound confirms TLF thickening in chronic Low Back Pain patients, correlating with pain intensity and dysfunction, though fascial stiffness may be a more sensitive marker in athletes. Reduced elasticity and shear motion arise from injury-related restrictions, altering local biomechanics and promoting persistent pain, potentially through systemic fascial involvement [10,11,12,13,14,15].

CN are fibro-adipose subcutaneous nodular formations, traditionally described as herniated adipose tissue through the superficial fascia, though recent evidence suggests they may represent well-demarcated fibro-adipose nodules or even lipomas [16,17]. These structures are often asymptomatic but have been described as biomechanical markers of motor dysfunction and muscle atrophy in patients with cLBP, particularly affecting paraspinal and gluteal musculature. Their hypermobility between fascial planes distinguishes them from normal adipose tissue and other soft tissue masses [16,17,18,19]. Ultrasound is the preferred modality for identifying Copeman nodule morphology, location, and mobility, while MRI helps delineate their relationship with surrounding muscles and fascia and excludes differential diagnoses [20,21]. In this perspective, the primary outcome of our study was to analyze the prevalence of the ultrasound marker triad (CN + TLF thickening > 3 mm + enthesophytes) in patients with cLBP. Secondary outcome was to study the possible correlation between ultrasound markers, pain intensity (NPRS) and between neuropathic pain (DN4).

## 2. Materials and Methods

### 2.1. Study Design and Population

This single-center, cross-sectional observational pilot study was conducted between 10 October 2024, and 10 October 2025. The study protocol was submitted, reviewed, and approved by the Ethics Committee of the University-Hospital Umberto I of Rome, Italy (protocol number 0495/2024, approval no. 7660, date 29 May 2024). Outpatients in the Physical and Rehabilitation Department of the Umberto I University-Hospital of Rome were screened and evaluated for inclusion. Patients with a confirmed diagnosis of cLBP, were enrolled in the study, whereas those with alternative potential sources of pain were excluded (e.g., lumbar radiculopathy (L5-S1), facet joint arthropathy, sacroiliac dysfunction, myofascial pain syndromes, piriformis syndrome, hip osteoarthritis, and gluteal tendinopathy). The study was conducted in accordance with the Declaration of Helsinki and in compliance with Good Clinical Practice guidelines. Results have been reported following the STROBE guidelines [22].

Inclusion criteria comprised (1) age 18–75 years; (2) cLBP (>12 weeks duration) localized to the SCN distribution territory (lateral lumbar and superior gluteal region); (3) pain intensity ≥ 4/10 on numeric pain rating scale (NPRS) at enrollment; (4) willingness to undergo ultrasound examination and complete pain assessment scales. Exclusion criteria included (1) systemic inflammatory or autoimmune diseases (e.g., spondyloarthritis, rheumatoid arthritis); (2) known malignancy, infection, or recent vertebral fracture; (3) clinical or imaging evidence of active lumbar radiculopathy; (4) pregnancy. Patients meeting inclusion criteria were enrolled. A control group of age- and sex-matched healthy volunteers without history of LBP or musculoskeletal disorders was recruited for comparison. The diagnosis of SCN entrapment was reached through a rigorous clinical process of exclusion. Patients were enrolled only after systematically ruling out other common causes of cLBP through negative provocative maneuvers for sacroiliac joint dysfunction, absence of radicular signs (L5-S1) on neurological examination, and negative findings for facet joint arthropathy or hip-related pain. The clinical suspicion was confirmed when symptoms were strictly confined to the SCN distribution and tenderness was identified over the posterior iliac crest (Valleix point), as described in the literature [3,23]. Musculoskeletal ultrasound was then utilized not for primary diagnosis, but to investigate the presence of surrogate biomechanical markers within this clinically identified population.

### 2.2. Clinical Assessment

Pain intensity was assessed using the Numeric Pain Rating Scale (NPRS) [24], ranging from 0 (no pain) to 10 (worst imaginable pain). Neuropathic pain component was evaluated using the Douleur Neuropathique 4 Questions (DN4) questionnaire, with scores ≥ 4/10 indicating neuropathic pain [25].

### 2.3. Ultrasound Assessment

All ultrasound examinations were performed by a single experienced operator blinded to participant clinical status, using a high-frequency 15 MHz linear transducer Sonosite FUJIFILM Edge (FUJIFILM Sonosite, Inc., Bothell, WA, USA). Participants were positioned prone with a cushion placed under the abdomen to reduce lumbar lordosis and facilitate probe access. The optimal scanning site was identified just above the posterior iliac crest, paramedian to the L5 transverse process, approximately 7–8 cm lateral to the midline. At this anatomical location, the TLF inserts onto the iliac crest, forming an osteo-fibrous tunnel through which the medial branches of the SCN course—the most frequent site of entrapment. The probe was positioned in the transverse plane, allowing identification of three key landmarks: the hyperechoic TLF, underlying paraspinal muscles (multifidus and erector spinae), and the iliac crest as an osseous reference. All measurements were performed applying minimal and constant probe pressure to avoid tissue distortion. For each parameter, three consecutive measurements were recorded, and the mean value was used for analysis to minimize intra-measurement variability. Ultrasound settings were optimized for each subject to ensure optimal contrast at the fascial–bone interface at the iliac insertion site.

### 2.4. Measured Parameters

**Figure 1:**
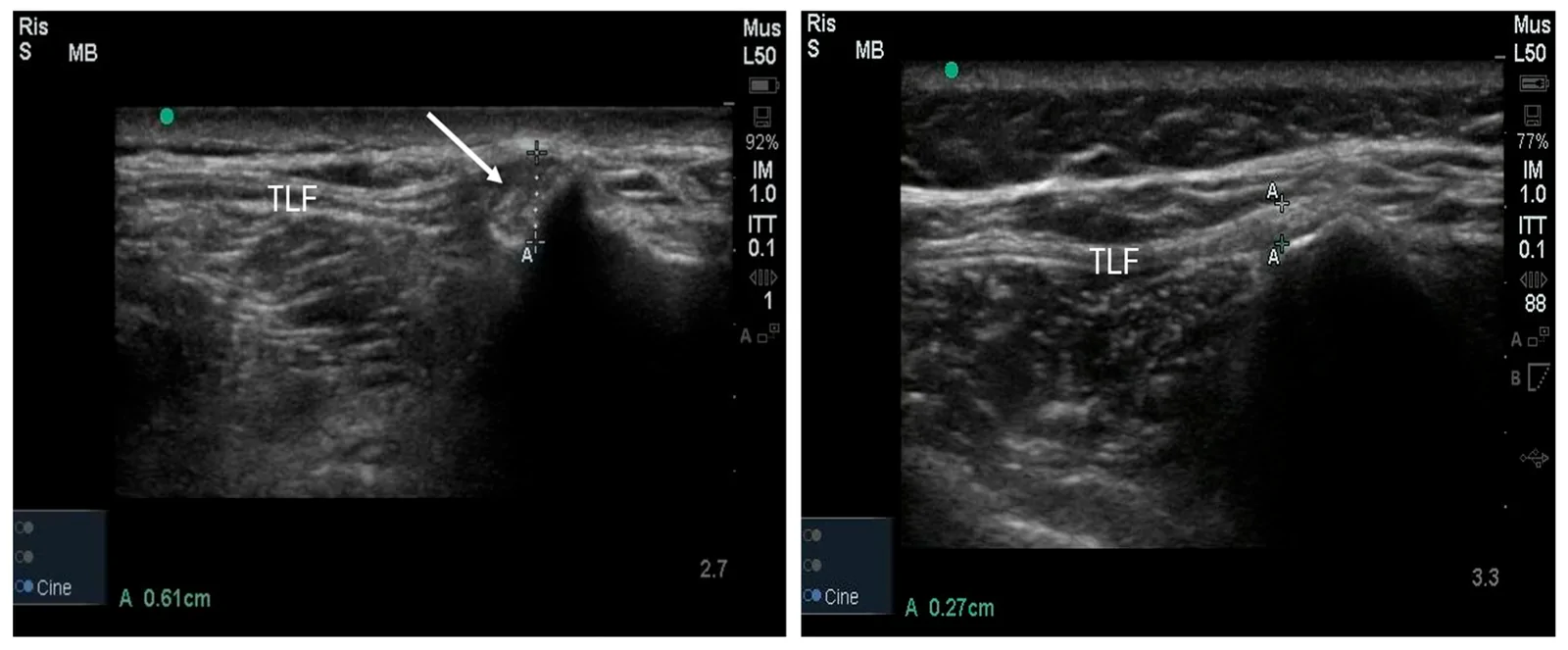
Ultrasound comparison of the thoracolumbar fascia (TLF) in short-axis view. On the left, pathological findings show evident thickening (white arrow) at the insertion site on the posterior iliac crest; on the right, normal TLF appearance in a healthy control without thickening. TLF: thoracolumbar fascia. The letter A represents the electronic calipers used to measure the thickness of the thoracolumbar fascia (TLF).

**Figure 2:**
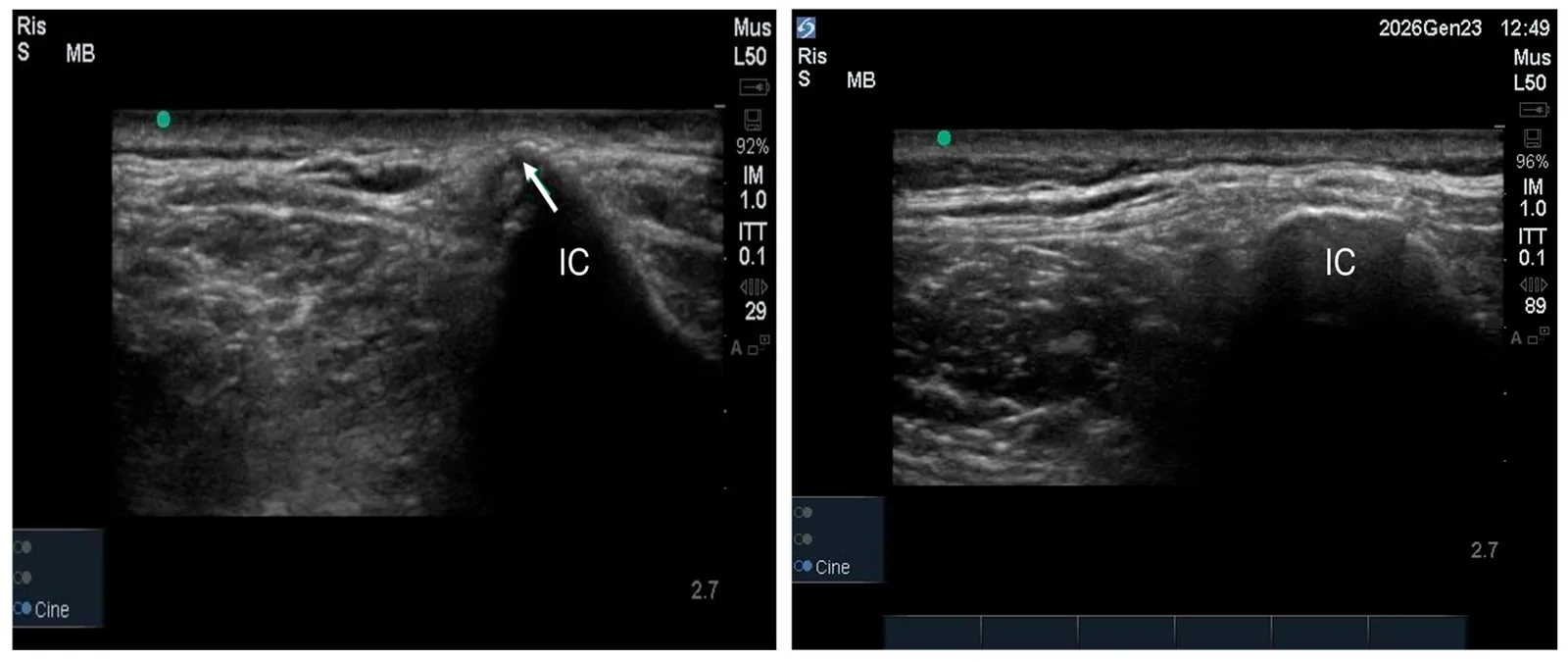
Ultrasound image of the iliac insertion site in short-axis view. The left side shows the presence of an iliac crest enthesophyte (white arrow) at the TLF insertion site; the right side shows a normal osseous profile of the iliac crest in a healthy control. IC: iliac crest.

**Figure 3:**
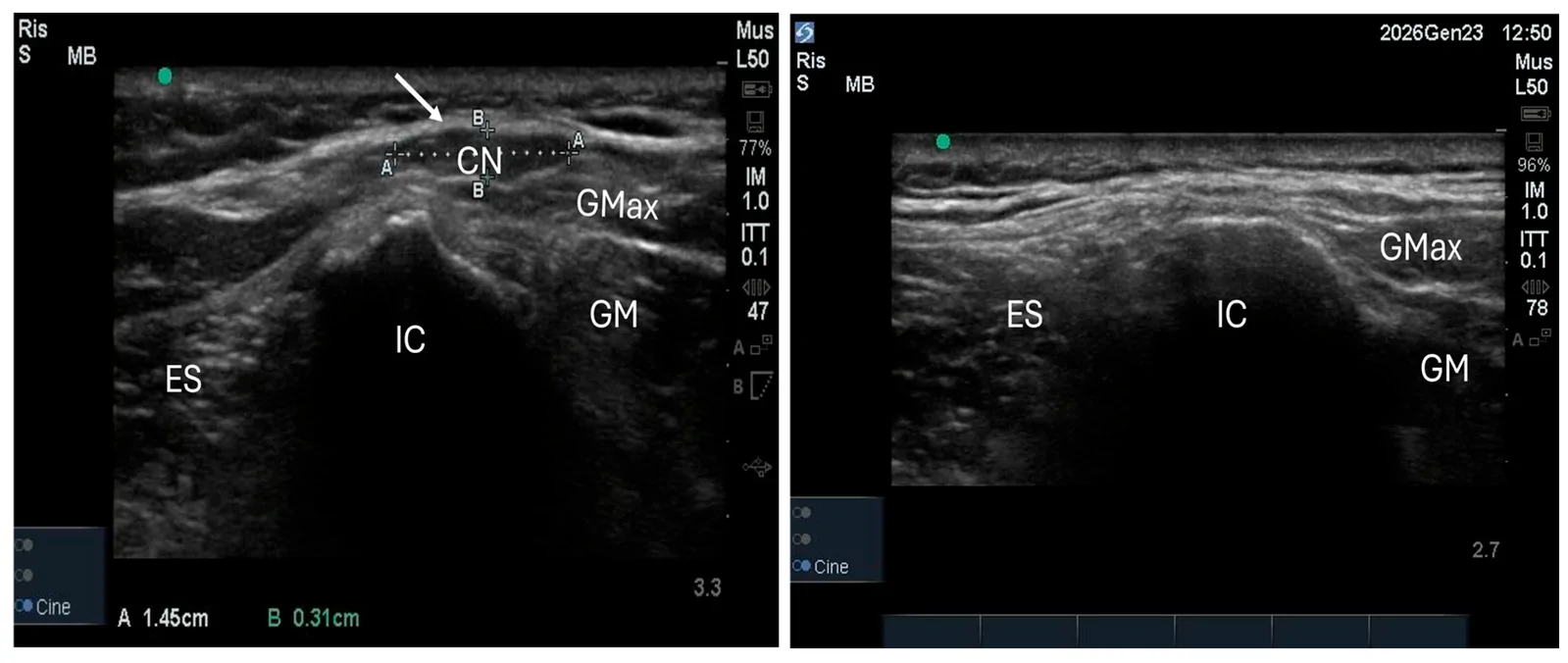
Ultrasound image of Copeman’s nodule (CN) in long-axis view. Identification of a CN (white arrow) in the lumbar region on the left, while the nodule is absent in the healthy control on the right. IC: iliac crest; GM: gluteus medius; Gmax: gluteus maximus; ES: erector spinae; CN: Copeman’s nodule. The letters A and B indicate the electronic calipers used for the dimensional assessment of the Copeman’s Nodule (CN).

Measured parameters included the following:

- TLF thickness (mm) measured in both longitudinal and transverse planes at the iliac insertion site. The selection of the measurement site—at the fascial insertion on the posterior iliac crest, specifically at the transition point where the fascia originates from the bone—represents a methodological divergence from previous studies and may account for the consistently higher thickness values observed. The exact measurement site was defined as the entheseal transition zone where the TLF fibers merge with the hyperechoic cortex of the posterior iliac crest. To ensure anatomical consistency, this point was identified 70–80 mm lateral to the L5 spinous process. Measurements were taken at the point of maximal fascial thickness before its complete osseous integration. This site was purposefully selected to establish a fixed anatomical landmark, ensuring a standardized and highly reproducible measurement that minimizes inter-individual variability. Moreover, focusing on this specific enthesis is clinically significant, as it corresponds to the osteo-fibrous tunnel where the medial branches of the SCN are most frequently entrapped (Figure 1).

- Iliac crest enthesophytes (calcific enthesopathy at the TLF insertion) (Figure 2).

- CN (hypoechoic subcutaneous fibro-adipose nodular formations) (Figure 3).

### 2.5. Statistical Analysis

Data normality was assessed using the Shapiro–Wilk test. Continuous variables were reported as mean ± standard deviation or median with interquartile range, depending on distribution. Categorical variables were reported as frequencies and percentages. Group comparisons: Mann–Whitney U test for continuous variables; Fisher exact test for categorical variables. Correlations: Spearman rank correlation coefficient (ρ) for non-parametric associations. Multivariate analysis: logistic regression assessed the predictive value of the ultrasound triad for high pain intensity. Statistical significance was set at *p* < 0.05. All analyses were performed using Jamovi (Version 2.4.11, The jamovi project, Sydney, Australia).

## 3. Results

### 3.1. Participant Characteristics and Clinical Profiles

The study enrolled twelve patients (mean age 51.2 ± 12.5 years) and twelve healthy controls (mean age 49.9 ± 9.3 years), with a balanced sex distribution (50% female) and no significant age difference (*p* = 0.76). Within the patient cohort, eight individuals had a history of cLBP previously attributed to sacroiliitis or facet joint syndrome that was refractory to conservative treatment. Additionally, two patients had developed persistent superior gluteal pain following lumbar fusion surgery, and two reported exacerbations during prolonged sitting or lumbar flexion.

### 3.2. Ultrasound Marker Prevalence and the “Marker Triad”

The prevalence of the ultrasound marker triad (CN, TLF thickness > 3 mm and enthesophytes) was significantly higher in the patient group compared to controls (91.7% vs. 8.3%, *p* < 0.001; Table 1). Specifically, Copeman nodules (CN) were identified in 91.7% of patients compared to only 8.3% of controls (*p* < 0.001). Interestingly, all CNs were located below the superficial fascia without evidence of herniation, challenging traditional definitions. Finally, iliac crest enthesophytes at the fascial insertion site were present in 58.3% of patients and entirely absent in the control group (*p* = 0.005), making them a highly specific marker for SCN entrapment.

### 3.3. TLF Thickness and Loss of Anisotropy

Mean TLF thickness was significantly greater in patients in both the longitudinal (3.53 ± 0.46 mm) and transverse (3.42 ± 0.39 mm) planes compared to controls (2.61 ± 0.28 mm and 2.50 ± 0.32 mm, respectively; *p* < 0.001 for both). To enhance diagnostic precision, we analyzed the discriminative power of a specific thickness threshold. While the literature often considers 2 mm as pathological, our data demonstrates that a 3 mm cut-off provides superior specificity. In our cohort, a threshold of 3 mm effectively separated the groups: the mean thickness in healthy controls plus one standard deviation (2.61 + 0.28 = 2.89 mm) remained below this value, whereas the patient group mean (3.53 mm) significantly exceeded it (*p* < 0.001). In total, 5 out of 12 patients (41.7%) demonstrated a TLF thickness strictly exceeding 3 mm (range 3.1–6.2 mm). In contrast, none of the healthy controls (0%) reached this value, as their maximum recorded thickness was 2.9 mm (mean 2.61 ± 0.28 mm). This confirms that a >3 mm cut-off serves as a highly specific surrogate marker for SCN entrapment. As noted previously, the choice to measure exactly at the fascial insertion site on the posterior iliac crest ensures a standardized landmark but also explains the higher absolute thickness values recorded in this study.

### 3.4. Correlation and Regression Analysis

Statistical analysis revealed a strong relationship between structural alterations and clinical symptoms. TLF thickness showed a strong positive correlation with pain intensity (NPRS), whereas no significant correlation was found with neuropathic pain scores (DN4), suggesting a predominantly nociceptive biomechanical mechanism. Furthermore, age was positively correlated with the presence of enthesophytes. Multivariate logistic regression confirmed that the marker triad accounted for 67% of the variance in NPRS scores (R^2^ = 0.67).

## 4. Discussion

The primary aim of our study was to analyze the prevalence of the ultrasound marker triad (CN + TLF thickening > 3 mm + enthesophytes) in patients with cLBP and the secondary one was to study the correlation between ultrasound markers, NPRS and DN4.

The results indicate that TLF thickening, combined with the CN and iliac enthesophytes, is significantly associated with nociceptive pain intensity in patients with suspected SCN entrapment. Importantly, this association does not extend to the neuropathic component assessed by DN4, suggesting a predominant role of nociceptive mechanisms mediated by fascial and musculoskeletal alterations.

### 4.1. Fascia as a Pain Generator

The TLF is richly innervated by Aδ and C nociceptive fibers. Structural alterations, particularly thickening and loss of elasticity, may induce peripheral sensitization responsible for nociceptive LBP. This pain is subjectively measurable via scales like the NPRS but does not necessarily reflect a neuropathic component detectable by DN4. However, this model alone cannot fully explain the well-localized pain in the SCN territory presenting clinical and pathophysiological features typical of nerve compression pain. Therefore, it is essential to distinguish between fascia-related LBP: predominantly nociceptive, related to fascial inflammation and mechanical stress, and SCN entrapment-related neuropathic pain, requiring a distinct diagnostic and therapeutic approach.

### 4.2. Biomechanical Microenvironment Hypothesis

We propose a pathophysiological model in which paraspinal and/or gluteal muscle atrophy reduces active spinal support, increasing passive TLF tension. This mechanical overload promotes fascial stiffening, increased traction on the posterior iliac crest, and compression of SCN branches coursing near the fascial insertion. Chronic mechanical stress may induce calcific enthesopathy (enthesophyte formation) and contribute to the appearance of CN—fibro-adipose subcutaneous formations frequently associated with muscle trophism changes. CN, often hypermobile and well-characterized on ultrasound, may function as adaptive cushioning elements modulating mechanical force transmission. Together, these findings delineate a biomechanical environment detectable by ultrasound that may predispose to or coexist with SCN entrapment syndrome. Although TLF convexity loss was observed in a high percentage of healthy controls (75%), its presence in isolation lacks diagnostic specificity. While convexity loss is common, we propose that TLF thickening > 3 mm is stronger primary marker. When integrated into the ‘ultrasound triad’ alongside CN and enthesophytes, it contributes to a highly specific biomechanical profile for SCN entrapment.

### 4.3. Clinical Implications

The identification of a reproducible ultrasound marker triad offers several clinical advantages: non-invasive diagnostic support complementing clinical examination; patient stratification for targeted rehabilitation or interventional treatment; monitoring treatment response through serial ultrasound assessment.

### 4.4. Therapeutic Considerations

Treatment must extend beyond symptomatic management (e.g., infiltrations, nerve blocks) to address the underlying biomechanical dysfunction. Key interventions include gluteal muscle strengthening, particularly gluteus maximus; progressive recruitment of erector spinae; targeted multifidus rehabilitation, as segmental atrophy is frequently associated with cLBP and impaired spinal stability; restoring neuromuscular control of the lumbo-pelvic-gluteal complex; these strategies aim to reduce nerve compression, limit fascial inflammation, and prevent progression of structural damage.

### 4.5. Study Strengths

Novel identification of a combined ultrasound marker triad; standardized ultrasound protocol and blinded operator; strong statistical correlations between markers and clinical pain.

### 4.6. Study Limitations

Our study is not without limitations. The primary limitation of this study is the small sample size (*n* = 24), which characterizes this work as a pilot study. While the findings are statistically significant, they should be interpreted with caution regarding generalizability. Additionally, the cross-sectional design precludes any causal inference between the identified ultrasound markers and the development of SCN entrapment. While ultrasound is inherently operator-dependent, we addressed this by employing a single experienced operator, thereby eliminating inter-operator variability. Finally, prospective validation in larger cohorts is essential to establish standardized ultrasound criteria and refine therapeutic protocols for this underrecognized cause of cLBP.

## 5. Conclusions

Musculoskeletal ultrasound reveals a reproducible marker triad for SCN entrapment, comprising CN, TLF thickening > 3 mm, and iliac enthesophytes. This non-invasive diagnostic approach demonstrates strong correlation with nociceptive pain intensity and may enhance clinical decision-making and guide targeted rehabilitation strategies. The observed ultrasound findings evoke the existence of a pathological biomechanical environment characterized by increased passive fascial tension, enthesopathic changes, and subcutaneous connective tissue alterations. This state, detectable through imaging, may both predispose to and perpetuate SCN entrapment syndrome. Prospective validation in larger cohorts is essential to establish standardized ultrasound criteria and refine therapeutic protocols for this underrecognized cause of cLBP. Future multicenter prospective studies with larger cohorts, standardized protocols, and longitudinal follow-up are needed to validate these findings and establish diagnostic thresholds.

## Figures and Tables

**Table 1:** Results of the analyses performed.

Statistical Category	Variable 1	Variable 2	Coefficient/Frequency	*p*-Value
Prevalence Comparison	Copeman Nodules (CN)	Group (Patients vs. Controls)	91.7% vs. 8.3%	<0.001
	Iliac Enthesophytes	Group (Patients vs. Controls)	58.3% vs. 0%	0.005
	TLF Thickness (>3 mm)	Group (Patients vs. Controls)	41.7% vs. 0%	<0.001
Dimensional Analysis	TLF Thickness (Long.)	Group (Patients vs. Controls)	3.53 mm vs. 2.61 mm	<0.001
	TLF Thickness (Trans.)	Group (Patients vs. Controls)	3.42 mm vs. 2.50 mm	<0.001
Correlation Analysis	TLF Thickness (Trans.)	Pain Intensity (NPRS)	Spearman ρ: 0.825	0.001
	Age	Iliac Enthesophytes	Spearman ρ: 0.666	0.018
	TLF Thickness	Neuropathic Pain (DN4)	Not Significant	>0.05
Multivariate Regression	Marker Triad Score	NPRS Variance		<0.001

## Data Availability

All data is available in the article.

## Abbreviations

The following abbreviations are used in this manuscript:

SCN	Superior Cluneal Nerve
cLBP	chronic Low Back Pain
CN	Copeman Nodules
TLF	Thoracolumbar Fascia
NPRS	Numeric Pain Rating Scale
DN4	Douleur Neuropathique en 4 questions
